# REALITIES AND CHALLENGES OF ELDER ABUSE REPORTED TO THE POLICE

**DOI:** 10.1093/geroni/igae098.1850

**Published:** 2024-12-31

**Authors:** Hitoshi Suda, Tadashi Wada, Kana Satoh, Tamiko Uehara

**Affiliations:** Seitoku University, Matsudo-city, Chiba, Japan; Hidamari Homecare Clinic, Chiba, Chiba, Japan; Seitoku University, Matsudo-city, Chiba, Japan; Matsudo City Medical Association, Matsudo City, Chiba, Japan

## Abstract

Nationwide, the number of elder abuse reports made to the police has increased. However, it was unclear if these reports resulted in an increase in cases substantiated as actual “elder abuse” by the police? This study used data from fiscal year 2019 in Matsudo City, analyzing a total of 171 elder abuse cases for this study. Among these, 73 cases were reported by the police, accounting for 42.7% of the total cases, and the perpetrators lived with the victims, indicating children were the most common perpetrators of 35 cases (47.9%), followed by spouse of 31 cases (42.5%), and others of 7 cases (9.6%). Out of the police-reported 73 cases, 60 cases (82.2%) were not substantiated as elder abuse under the Act on Prevention of Elder Abuse, and only 13 cases (17.8%) were substantiated as elder abuse. This outcome led us to the conclusion that the increase in police reports does not necessarily indicate an increase in the cases substantiated as “elder abuse,” and highlighted the limited scope of definitions of Elder Abuse in the Act. For example, despite cohabitation, the elder abuse cases where even actual elder violence occurs and requires hospitalization, are not considered as “elder abuse” if there is no caregiving relationship and many of such elder violence cases become to be called “situation-based violence”. It is strongly suggested that the definition of elder abuse of cohabitants with no caregiver relationships should be also included as “elder abuse” in accordance with WHO guidelines.

